# Expanding the spectrum of the giant panda sign: a bright-eye variant in Wilson disease

**DOI:** 10.1016/j.radcr.2026.06.055

**Published:** 2026-07-07

**Authors:** Srinidhi Srinivasan, Arun Paul, Jampana Sahitya, Swathi Mulupuru, Eswar K. Sakinala

**Affiliations:** a Department of Radiology, Alluri Sitaramaraju Academy of Medical Sciences, Eluru, Andhra Pradesh, India; b Department of Emergency Medicine, Viswabharathi Medical College, Kurnool, Andhra Pradesh, India; c Department of General Medicine, Alluri Sitaramaraju Academy of Medical Sciences, Eluru, Andhra Pradesh, India

**Keywords:** Giant panda sign, Bright eyes sign, Basal ganglia, Brain MRI, Midbrain, T2 hyperintensity, Hepatolenticular degeneration, Wilson disease

## Abstract

Wilson disease is an autosomal recessive disorder that affects copper metabolism due to mutations in the ATP7B gene. It causes problems with the liver and the nervous system. On magnetic resonance imaging (MRI), the typical sign is the “face of the giant panda” in the midbrain. A rarer sign, called the “panda with bright eyes,” suggests more extensive brainstem involvement and is not often seen. A 21-year-old man developed worsening behavior changes, mood swings, and trouble with his studies. He later had tremors in both arms and mild speech difficulties. His liver tests were abnormal, and his ceruloplasmin level was low at 8 mg/dL. An eye exam showed Kayser–Fleischer rings in both eyes. Brain MRI showed symmetrical T2/FLAIR hyperintensities in the caudate nuclei, putamina, globus pallidi, thalami, posterior limbs of the internal capsules, and both the superior and middle cerebellar peduncles, as well as the pons. Increased T2 signal in the red nuclei and substantia nigra produced the “panda with bright eyes” sign. There was also diffusion restriction in both globus pallidi and thalami. The Leipzig score confirmed Wilson disease. The patient started D-penicillamine treatment and showed clinical improvement. Radiologists need to recognize both common and rare MRI features of Wilson disease, especially when neuropsychiatric symptoms are present. Spotting the “panda with bright eyes” sign can help with early diagnosis, guide metabolic testing, and allow prompt chelation therapy, which can improve neurological outcomes.

## Introduction

Wilson disease is a rare autosomal recessive disorder of copper metabolism caused by mutations in the ATP7B gene located long arm of chromosome 13, resulting in defective hepatic copper excretion and subsequent accumulation of copper in various organs, predominantly the liver, central nervous system, and eyes [[1], [2], [3], [4]]. Clinical presentation is heterogeneous and varies with age, with hepatic manifestations commonly seen in children and adolescents, while neurological and psychiatric manifestations predominate in young adults.

Neuropsychiatric manifestations include behavioral changes, personality disturbances, tremors, dystonia, and cognitive decline, often mimicking primary psychiatric or neurological disorders and leading to diagnostic delay. The presence of Kayser–Fleischer rings and characteristic neuroimaging findings, such as the “face of the giant panda” sign on magnetic resonance imaging, are highly suggestive of the diagnosis. [5] However, rarely it can manifest as panda with “ bright eyes” sign which interestingly is different from signal intensity seen in face of giant panda sign. Early recognition is crucial, as Wilson disease is a treatable condition, and timely initiation of chelation therapy can halt disease progression and improve outcomes. We report a case of a 21-year-old male presenting with prominent neuropsychiatric symptoms and altered liver function tests, subsequently diagnosed with Wilson disease.

## Case presentation

A 21-year-old male presented with complaints of progressive behavioral changes over the preceding several months. According to his family, the patient developed increased irritability, mood swings, social withdrawal, and decline in academic performance, which were markedly different from his baseline personality. He also exhibited psychiatric manifestations, including emotional instability and inappropriate behavior, for which he initially sought psychiatric consultation. Over the following weeks, the patient developed tremors involving both hands, more pronounced during voluntary movements and fine motor tasks such as writing and eating. The tremors were insidious in onset and gradually progressive. There was no history of seizures, loss of consciousness, focal neurological deficits, headache, or visual disturbances. The patient reported generalized fatigue and reduced appetite but denied jaundice, abdominal distension, hematemesis, melena, or pedal edema. There was no history of alcohol intake, illicit drug use, or exposure to hepatotoxic medications. Past medical history was unremarkable, and there was no similar illness in family members. Preliminary investigations elsewhere revealed altered liver function tests, prompting referral for further evaluation.

On general physical examination, the patient was conscious and oriented, with stable vital signs. Mild scleral icterus was noted. There were no stigmata of chronic liver disease such as spider nevi, palmar erythema, or ascites. Slit-lamp examination revealed bilateral Kayser–Fleischer rings. Neurological examination revealed bilateral upper limb tremors, predominantly action and postural tremors. Mild dysarthria was noted. Muscle tone and power were normal in all limbs, with intact deep tendon reflexes. No focal motor or sensory deficits were identified. Gait examination showed mild unsteadiness. Psychiatric evaluation revealed behavioral abnormalities with impaired judgment and emotional lability. Abdominal examination revealed no hepatosplenomegaly. Cardiovascular and respiratory system examinations were within normal limits.

Magnetic resonance imaging of the brain demonstrated bilateral symmetrical T2/FLAIR hyperintensities in head of caudate nucleus, lateral aspect of bilateral putamen, posterior limbs of internal capsules, lateral aspect of bilateral thalami, superior and middle cerebellar peduncles and pons. There is symmetrical hyperintensity in bilateral globus pallidi. There is relative T2 hyperintensity in red nucleus and substantia nigra giving “ bright eyes of panda” sign, which is hypo intense on FLAIR giving “ face of giant panda” sign. On diffusion weighted imaging, there is diffusion restriction seen in bilateral red nuclei, globus pallidi and thalami, with no restriction seen in bilateral putamen (Fig. 1, Fig. 2, Fig. 3, Fig. 4, Fig. 5).

**Fig. 1:**
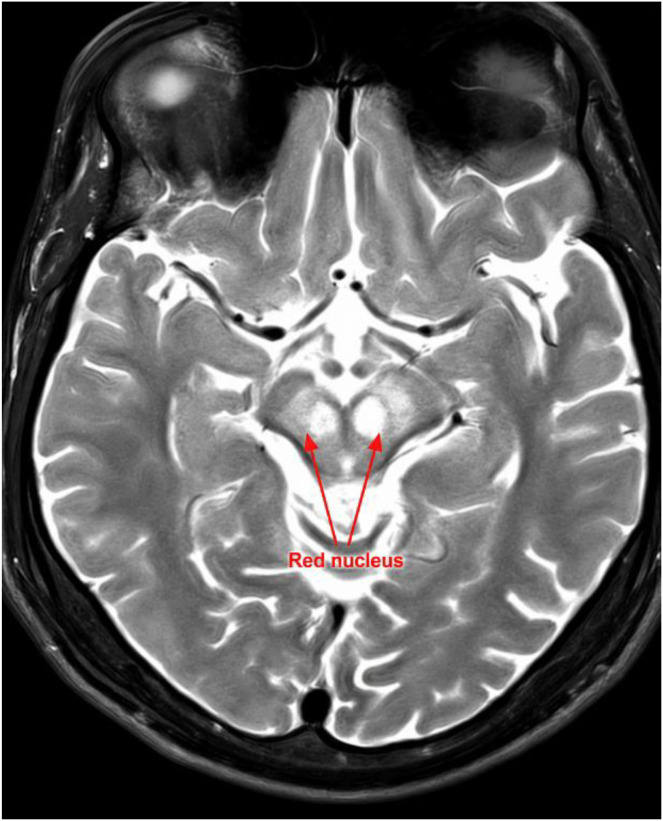
Axial T2-weighted MRI brain demonstrates bilateral symmetrical T2 hyperintense signal changes centered in the red nuclei of the midbrain. Sequence/distribution: Axial T2W sequence; bilateral red nuclei/midbrain involvement.

**Fig. 2:**
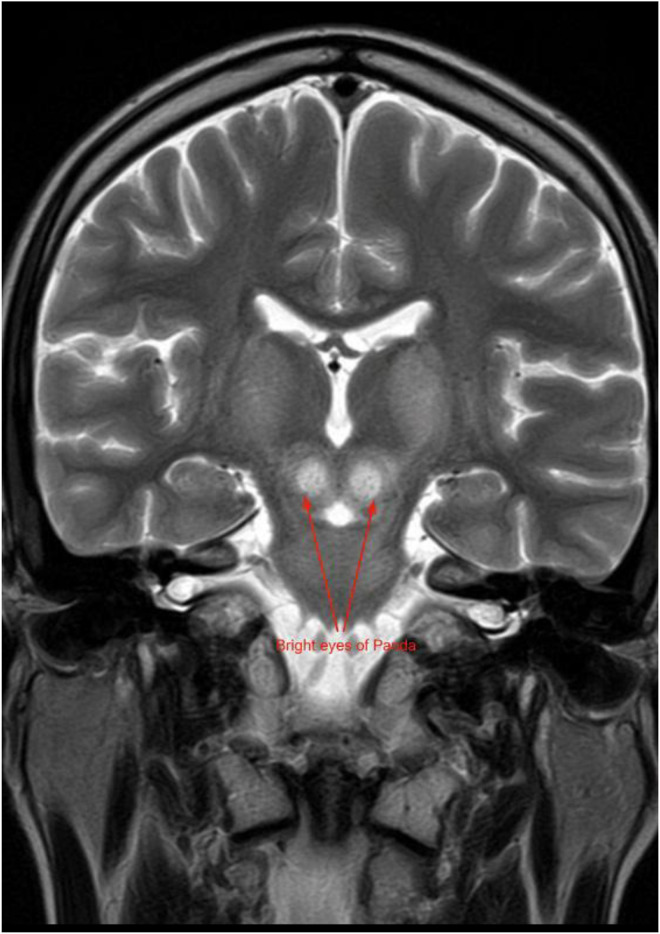
Coronal T2-weighted MRI brain demonstrates bilateral symmetrical hyperintense signal changes involving the red nuclei, producing the characteristic “bright eyes of the panda” appearance. Sequence/distribution: Coronal T2W sequence; bilateral red nuclei/midbrain involvement.

**Fig. 3:**
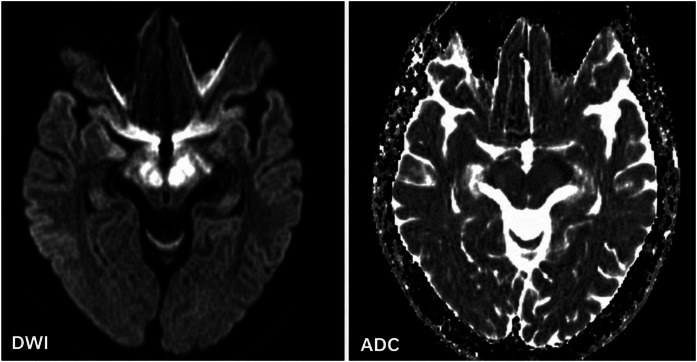
Diffusion-weighted image and corresponding ADC map demonstrate bilateral symmetrical diffusion restriction involving bilateral red nuclei and substantia nigra appearing hyperintense on DWI and hypointense on ADC, consistent with true restricted diffusion. Sequence/distribution: DWI with ADC correlation; bilateral red nuclei showing restricted diffusion.

**Fig. 4:**
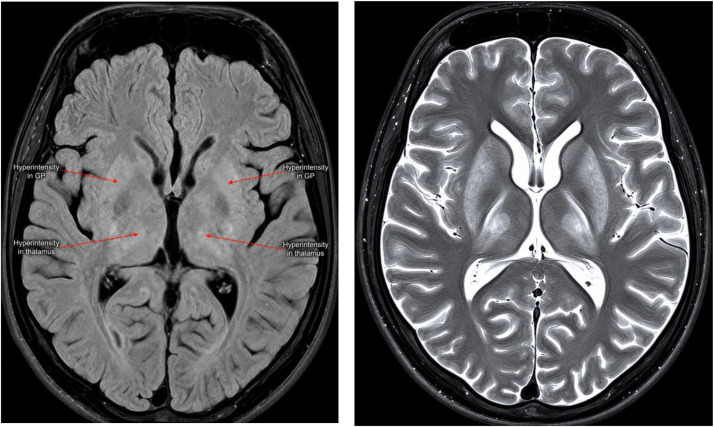
Axial FLAIR and T2-weighted MRI brain demonstrate bilateral symmetrical hyperintense signal changes involving the globus pallidi, putamina and caudate nuclei, with central hypointense signal within the globus pallidi. Similar symmetrical hyperintense signal changes are noted in the bilateral thalami. Sequence/distribution: Axial FLAIR and T2W sequences; bilateral basal ganglia and thalamic involvement.

**Fig. 5:**
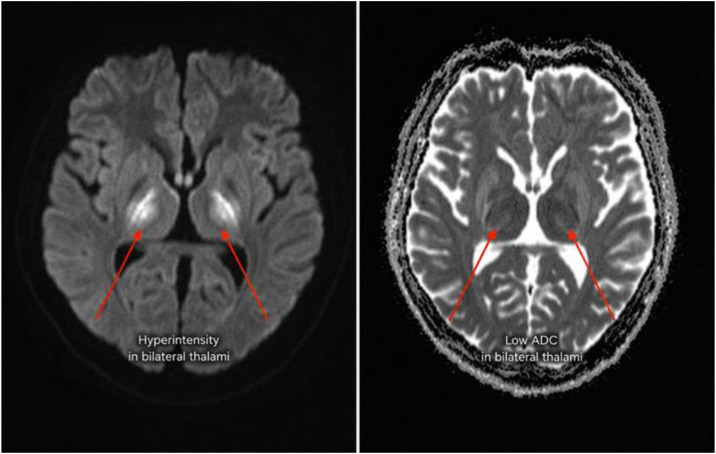
Diffusion-weighted image and corresponding ADC map demonstrate bilateral symmetrical diffusion restriction involving the thalami, appearing hyperintense on DWI and hypointense on ADC, suggestive of acute cytotoxic injury in Wilson disease. Sequence/distribution: DWI with ADC correlation; bilateral thalamic restricted diffusion.

Laboratory investigations showed elevated serum transaminases and a markedly reduced serum ceruloplasmin level of 8 mg/dL. Twenty-four-hour urinary copper excretion was elevated (>100 µg/d), supporting increased body copper burden.

Based on the Leipzig scoring system, the patient had a cumulative score of 6 (serum ceruloplasmin <10 mg/dL, neurological involvement, and elevated urinary copper excretion), strongly diagnostic of Wilson disease. The diagnosis was further supported by characteristic neuroimaging findings and clinical improvement following initiation of chelation therapy with D-penicillamine.

## Discussion

Wilson disease is a rare inherited disorder affecting copper metabolism. Its clinical presentation varies, often involving hepatic, neurological, and psychiatric systems. Hepatic symptoms are more common in children, while neurological and psychiatric features predominate in adolescents and young adults [6]. Our patient showed involvement of all 3 systems, leading to early consideration of a metabolic cause.

Neuroimaging is important for diagnosing Wilson disease. On T2-weighted MRI, the classic “face of the giant panda” sign can be seen in the midbrain. This sign shows up as a bright tegmentum, dark red nuclei, and normal lateral substantia nigra, making the area look like a panda’s face. This pattern reflects copper buildup in the tegmentum, which causes demyelination, gliosis, and symmetrical T2 changes [[7], [8], [9]].

Our patient exhibited the less common “panda with bright eyes” sign, which is a variation of the “face of the giant panda” sign. This happens when the red nuclei look bright on T2-weighted images, suggesting the disease is progressing. The basal ganglia, particularly the putamen, and sometimes the caudate and globus pallidus, can also be affected. In this sign, the red nuclei are brighter than the rest of the midbrain on T2 MRI [10]. Our patient’s scans showed this rare pattern. T2 hyperintensity of the red nuclei has also been described by Kallollimath et al. [8] and Sarapura-Castro et al. [9] as a rare variant of the giant panda sign in Wilson disease.

Besides midbrain changes, our patient’s T2 MRI showed bright signals in both putamina, which is a common early sign of Wilson disease. As the disease progresses, the caudate nucleus and globus pallidus can also be affected [11]. In children and adolescents, thalamic involvement can be caused by other metabolic or mitochondrial disorders. For example, Leigh syndrome also shows symmetrical involvement but is identified by a lactate peak on MR spectroscopy, which was not seen in our patient [12,13]. Other possible diagnoses include hypoxic-ischemic injury, organic acidemias, toxic encephalopathies, Menkes disease, aceruloplasminemia, and manganese toxicity. These are usually distinguished by the clinical situation and lab findings [10].

Hypoxic–ischemic injury happens suddenly after a known lack of oxygen. It shows restricted diffusion in the basal ganglia on MRI and does not progress over time, which is different from the gradual course of Wilson disease.

Organic acidemias usually appear in infants or young children with episodes of metabolic crisis, such as metabolic acidosis, high ammonia, and abnormal organic acids in urine. These symptoms do not occur in Wilson disease [14].

Toxic encephalopathies, like those from carbon monoxide or methanol, affect the basal ganglia depending on the toxin involved. They are diagnosed based on a history of exposure, sudden onset, and lab results [15].

Menkes disease appears in infancy with low copper and ceruloplasmin levels. Aceruloplasminemia shows T2 dark iron deposits and other body symptoms, while manganese toxicity causes T1 bright signals in the pallidum. In our patient, low ceruloplasmin, high urinary copper, and T2 bright spots in the basal ganglia and midbrain pointed to Wilson disease. Even though imaging findings can overlap, differences in when symptoms start, how the disease progresses, and metabolic results help tell these conditions apart [16].

We confirmed the diagnosis by finding low blood ceruloplasmin and high urinary copper, which met the Leipzig scoring system criteria. Although we did not do genetic testing for the ATP7B mutation, the clinical, lab, and imaging findings strongly supported Wilson disease.

This case stands out because it shows both classic and rare MRI signs on T2, FLAIR, and diffusion-weighted images, along with involvement of multiple systems. These rare imaging features are not often reported and may point to more severe or advanced disease [17]. This highlights the need for careful MRI interpretation.

We started treatment with D-penicillamine and zinc to help remove copper. The patient’s neurological symptoms got better over 3 to 4 months. At follow-up, we saw partial recovery, but long-term imaging results are still pending. Early diagnosis and chelation are crucial to prevent lasting brain damage [18,19]. This case shows the importance of combining clinical, lab, and imaging data to diagnose Wilson disease and report rare MRI findings accurately.

## Conclusion

The purpose of this case report is to highlight the spectrum of clinicoradiological features of Wilson disease to make the readers aware of myriad of imaging features apart from the famous face of giant panda sign to make the essential diagnosis and provide further management before progression to extensive involvement of brain stem and basal ganglia.

## Patient consent

Written informed consent was obtained from the patient’s parents for the publication of this study and accompanying images.

## References

[1] Członkowska A, Litwin T, Dusek P, Ferenci P, Lutsenko S, Medici V (2018). Wilson disease. Nat Rev Dis rPrimers.

[2] Ferenci P, Caca K, Loudianos G, Mieli-Vergani G, Tanner S, Sternlieb I (2003). Diagnosis and phenotypic classification of Wilson disease. Liver Int.

[3] Mulligan C., Bronstein J.M. (2020). Wilson disease: an overview and approach to management. Neurol Clin..

[4] Guindi M. (2019). Wilson disease. Semin Diagn Pathol..

[5] Prashanth L.K., Sinha S., Taly A.B., Vasudev M.K. (2010). Do MRI features distinguish Wilson's disease from other early onset extrapyramidal disorders? An analysis of 100 cases. Mov Disord..

[6] Roberts E.A., Schilsky M.L., Adam M.P., Mirzaa G.M., Pagon R.A., Wallace S.E., Bean L.J.H., Gripp K.W. (2023). GeneReviews®.

[7] Medscape. Wilson Disease – Practice Essentials, Background, Etiology. eMedicine. https://emedicine.medscape.com/article/183456-overview#a1. Updated June 25, 2026.

[8] Castro E.S., Quiñones J.R., Olivas M.C. (2020). Panda with ``bright eyes'': a rare sign in Wilson disease. Arq Neuropsiquiatr.

[9] Kallollimath P., Nagappa M., Sinha S., Saini J., Bindu P.S., Taly A.B. (2013). Panda with ``bright eyes'' in Wilson's disease. Neurol India..

[10] Litwin T., Ogrodnik B.R., Antos A., Przybyłkowski A., Członkowska A., Bembenek J.P. (2024). 11.Brain magnetic resonance imaging in Wilson's disease: significance and practical aspects. A narrative review. Brain Sci..

[11] Ranjan A., Kalita J., Kumar S., Bhoi S.K., Misra U.K. (2015). A study of MRI changes in Wilson disease and its correlation with clinical features and outcome. Clin Neurol Neurosurg..

[12] Sijens P.E., Smit G.P.A., Rodiger L.A., van Spronsen F.J., Oudkerk M., Rodenburg R.J. (2008). MR spectroscopy of the brain in Leigh syndrome. Brain Dev..

[13] Baertling F, Rodenburg RJ, Schaper J, Smeitink JA, Koopman WJ, Mayatepek E, Morava E, Distelmaier F (2014). A guide to diagnosis and treatment of Leigh syndrome. J Neurol Neurosurg Psychiatry.

[14] Häberle J. (2021). Hyperammonemia in inherited metabolic diseases. Nutrients..

[15] Arora V, Nijjar IBS, Multani AS, Singh JP, Abrol R, Chopra R (2007). MRI findings in methanol intoxication: A report of two cases. Br. J. Radiol..

[16] Thirunavukkarasu S., Nair P.P., Wadwekar V. (2013). Acute bilateral putaminal haemorrhagic necrosis in methanol poisoning. BMJ Case Rep..

[17] Hermann W. (2019). Classification and differential diagnosis of Wilson's disease. Ann Transl Med..

[18] Ogrodnik B.R., Litwin T., Antos A., Przybyłkowski A., Członkowska A., Bembenek J.P. (2022). Neurological worsening in patients with Wilson disease. J Clin Med..

[19] Kozić D, Svetel M, Petrović B, Dragasević N, Semnic R, Kostić VS (2003). MR imaging of the brain in patients with hepatic form of Wilson’s disease. Eur J Neurol.

